# Development of Xanamem, an isoform 1 specific inhibitor of 11‐βHSD, as a procognitive and disease modifying treatment for mild/moderate Alzheimer’s disease. A pharmacodynamic and biomarker driven approach

**DOI:** 10.1002/alz.089127

**Published:** 2025-01-09

**Authors:** Paul Rolan, Jack Taylor, Jonathan Seckl, John E Harrison, Christopher Li‐Hsian Chen, Colm Farrell, Paul Maruff, Michael Woodward, Dana Hilt

**Affiliations:** ^1^ Actinogen Medical, Sydney Australia; ^2^ Actinogen Medical, Sydney, ACT Australia; ^3^ University of Edinburgh, Edinburgh United Kingdom; ^4^ Scottish Brain Sciences, Edinburgh, Scotland United Kingdom; ^5^ Metis Cognition Ltd., Kilmington United Kingdom; ^6^ Memory Ageing and Cognition Centre, National University Health System, Singapore Singapore; ^7^ ICON Inc, Dublin Ireland; ^8^ Cogstate Ltd., Melbourne, VIC Australia; ^9^ Florey Institute of Neuroscience and Mental Health, Parkville, VIC Australia; ^10^ Austin Health, Melbourne, VIC Australia

## Abstract

**Background:**

Selecting the optimal dose for clinical development is especially problematic for drugs directed at CNS‐specific targets. For drugs with a novel mechanism of action, these problems are often greater. We describe Xanamem’s clinical pharmacology, including the approach to dose selection and proof‐of‐concept studies. Xanamem is an isoform 1 specific inhibitor of 11‐βHSD which acts to reduce the tissue production of cortisol. Xanamem is under clinical development as a pro‐cognitive and disease modifying drug for Alzheimer’s disease as well as other neurological and psychiatric diseases involving CNS dysregulation of cortisol. Elevation of CNS cortisol has been associated with impaired cognition, neuroinflammation, and neuronal death.

**Method:**

Clinical pharmacology analyses included pharmacokinetic and pharmacodynamic data from SAD and MAD dose trials in healthy volunteers; population pharmacokinetics; cognition from patients in a phase 2a trial in Alzheimer’s disease; multiple dose PET target occupancy study to determine target inhibition in the brain in healthy elderly participants and Alzheimer’s patients; two phase 1b trials in healthy older participants that assessed safety; and pharmacodynamic endocrinology and quantitative cognitive parameters.

**Result:**

Xanamem demonstrated high CNS target occupancy at all doses studied by PET (5‐30 mg/day). At doses of 5 to 70 mg/day ACTH levels approximately doubled at steady‐state. After first doses, plasma cortisol was slightly reduced but was not decreased at steady‐state in doses of ≤ 10mg/day but was within normal levels. In three trials Xanamem demonstrated pro‐cognitive effects, primarily on attention and working memory (5‐20 mg/day). Xanamem also demonstrated potentially disease modifying treatment effects in mild AD in patients with biomarker positive AD (elevation of plasma pTau181) as shown by decrease in the rate of CDR‐SB worsening over 12 weeks (10 mg/day) versus placebo.

**Conclusion:**

This series of trials demonstrates that the optimal dose of Xanamem can be selected based on CNS PET occupancy data coupled with pharmacodynamic endocrinology and quantitative cognition testing. Pro‐cognitive and potentially disease modifying effects were observed at doses consistent with high CNS target occupancy. Xanamem is now in larger Phase 2 clinical outcome trials in MDD and mild/mod AD.

